# Supplementary figures and images for: Substrate-Driven Mapping of the Degradome by Comparison of Sequence Logos
Source: PLoS Comput Biol. 2013 Nov 14;9(11):e1003353. doi: 10.1371/journal.pcbi.1003353 (PMC3828135; doi:10.1371/journal.pcbi.1003353)

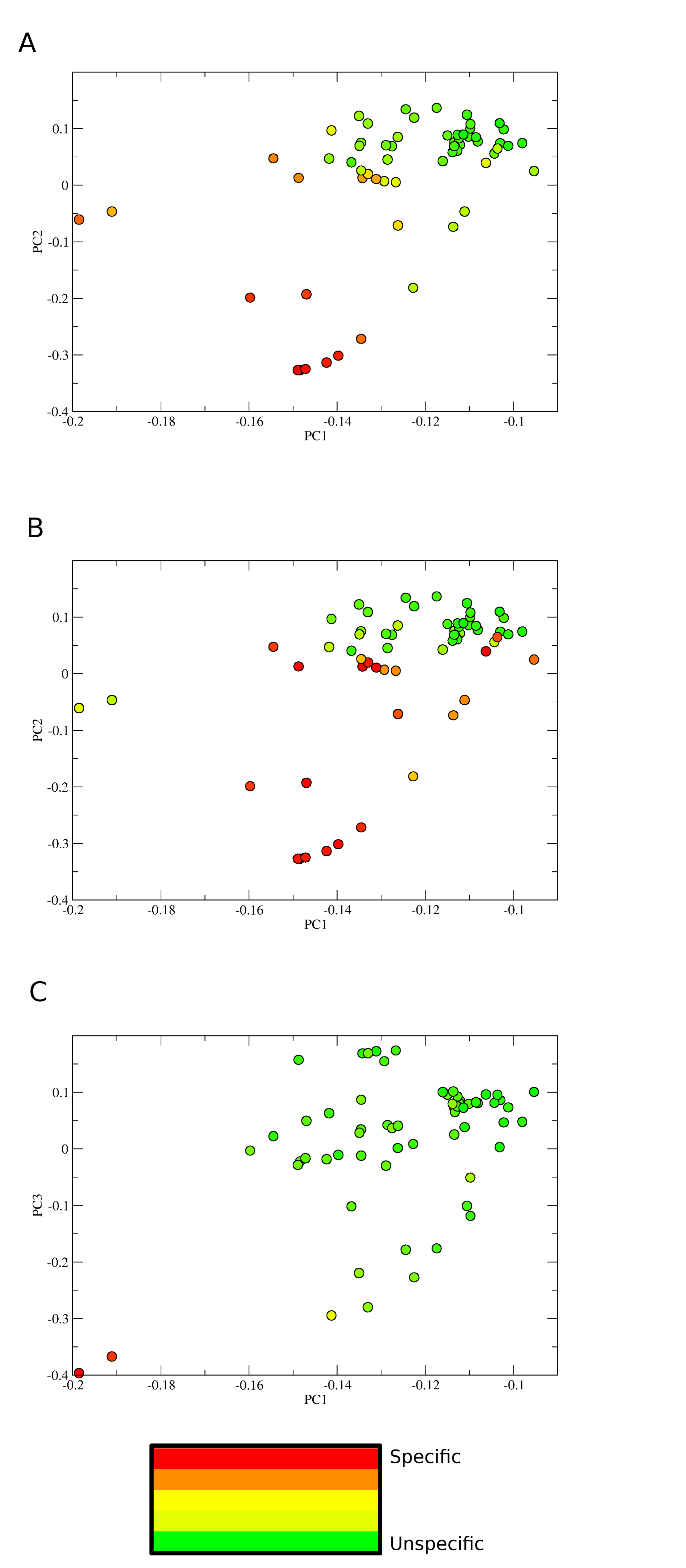

Supplement: Figure S1 — Principal component analysis of the protease distance matrix: Proteases mapped to the lower-dimensional degradome map from principal component analysis are colored according to their subpocket-wise cleavage entropies in a color range from red (specific) to green (unspecific). Proteases are colored by subpocket-wise cleavage entropy over pockets in the non-prime region (S4 to S1) in figure S1a against principal components 1 and 2. Figures S1b and S1c show a coloring according to subpocket-wise cleavage entropy of pockets S1 and S3′ respectively in a scatter plot of principal components 1, 2 and 3. (TIFF) [file pcbi.1003353.s001.tiff]
