# Supplementary material for: Substrate-Driven Mapping of the Degradome by Comparison of Sequence Logos
Source: PLoS Comput Biol. 2013 Nov 14;9(11):e1003353. doi: 10.1371/journal.pcbi.1003353 (PMC3828135; doi:10.1371/journal.pcbi.1003353)
Supplement: Table S1 — List of 62 investigated proteases annotated with MEROPS and ChEMBL identifiers as well as catalytic types, number of substrates and sequence logo. Proteases are sorted according to MEROPS identifier to assure grouping of evolutionary branches. (PDF) [file pcbi.1003353.s002.pdf]

Protease	MEROPS	ChEMBL	Catalytic Type	Substrates	Sequence Logo
Pepsin A	A01.001	3295	Aspartic	417	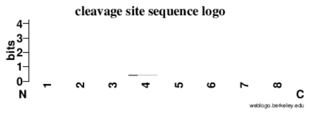
CathepsinD	A01.009	2581	Aspartic	741	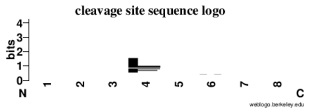
Cathepsin E	A01.010	3092	Aspartic	1300	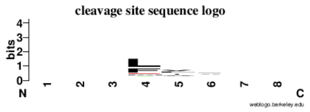
Rhizopuspepsin	A01.012	4253	Aspartic	249	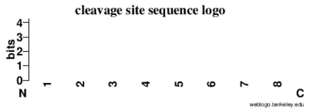
Aspergillopepsin	A01.016	---	Aspartic	159	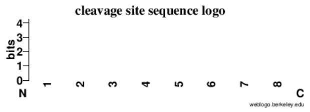
Necepsin 1	A01.053	---	Aspartic	134	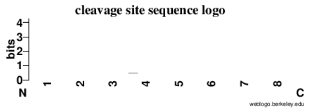
HIV-1 Retropepsin	A02.001	243	Aspartic	1049	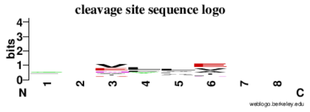
Cathepsin L	C01.032	3837	Cysteine	1090	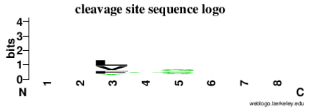
Cathepsin L1	C01.033	5291	Cysteine	196	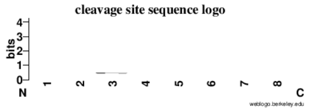
Cathepsin S	C01.034	2954	Cysteine	731	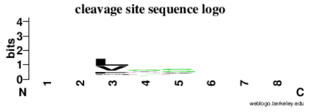
Falcipain 2	C01.046	3488	Cysteine	152	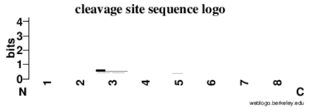
Cathepsin B	C01.060	4072	Cysteine	634	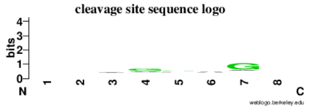
Falcipain 3	C01.063	4510	Cysteine	126	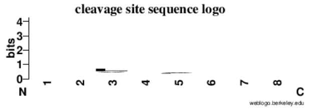
Cathepsin K	C01.136	268	Cysteine	182	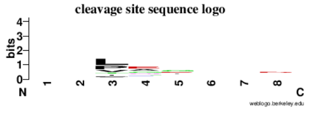

Protease	MEROPS	ChEMBL	Catalytic Type	Substrates	Sequence Logo
Calpain 1	C02.001	3891	Cysteine	102	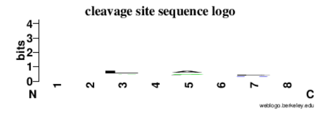
Calpain 2	C02.002	2382	Cysteine	164	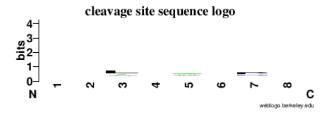
Caspase 1	C14.001	4801	Cysteine	175	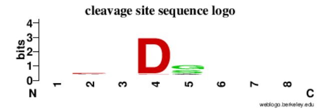
Caspase 3	C14.003	2334	Cysteine	625	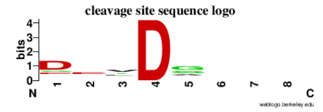
Caspase 7	C14.004	3468	Cysteine	169	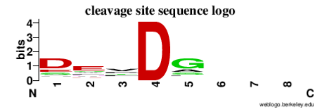
Caspase 6	C14.005	3308	Cysteine	201	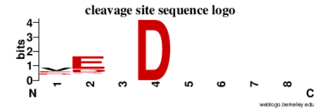
Thimet Oligopeptidase	M03.001	---	Metallo	124	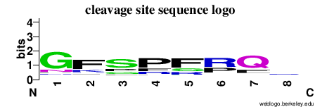
Neurolysin	M03.002	---	Metallo	123	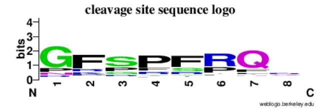
Thermolysin	M04.001	3392	Metallo	280	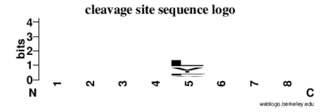
MMP 8	M10.002	4588	Metallo	114	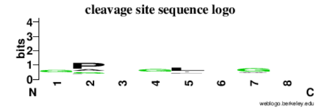
MMP 2	M10.003	333	Metallo	3429	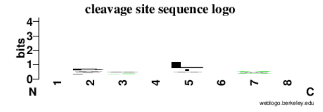
MMP 9	M10.004	321	Metallo	367	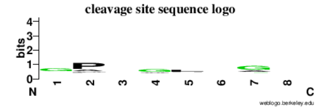
MMP 3	M10.005	283	Metallo	179	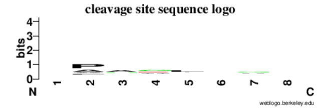

Protease	MEROPS	ChEMBL	Catalytic Type	Substrates	Sequence Logo
MMP 7	M10.008	4073	Metallo	192	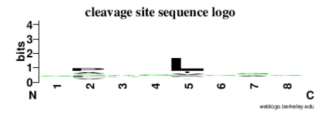
MMP 12	M10.009	4393	Metallo	218	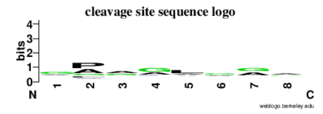
MMP 13	M10.013	280	Metallo	147	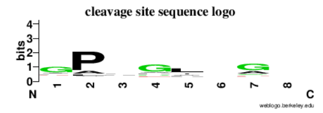
Membrane-MMP 1	M10.014	332	Metallo	129	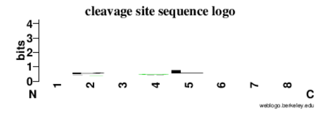
Astacin	M12.001	---	Metallo	209	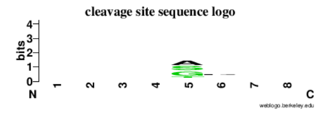
Meprin	M12.002	---	Metallo	776	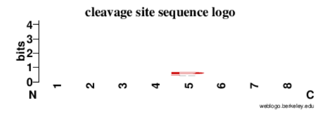
LAST MAM Peptidase	M12.033	---	Metallo	433	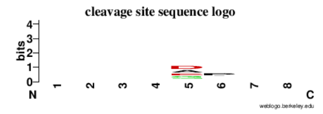
Neprilysin	M13.001	1944	Metallo	122	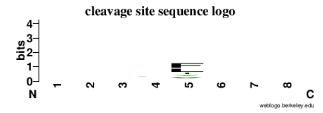
Peptidyl-Lys Metallopeptidase	M35.004	---	Metallo	2106	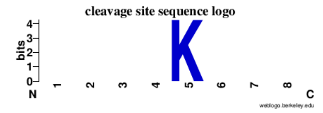
Chymotrypsin A	S01.001	3314	Serine	1061	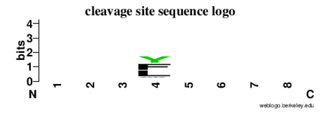
Granzyme B	S01.010	2316	Serine	1255	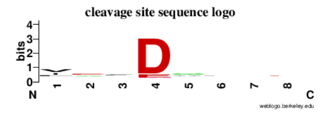
Elastase 2	S01.131	248	Serine	482	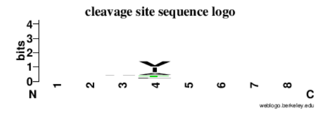
Cathepsin G	S01.133	4071	Serine	468	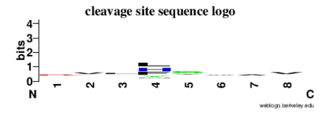
Granzyme A	S01.135	4307	Serine	280	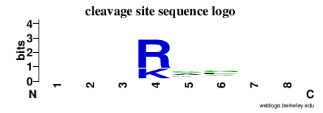

Protease	MEROPS	ChEMBL	Catalytic Type	Substrates	Sequence Logo
Granzyme B rodent	S01.136	---	Serine	177	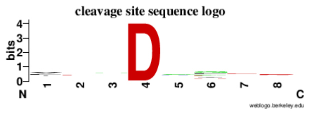
Chymase	S01.140	4068	Serine	106	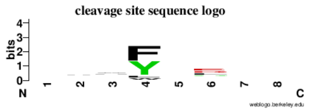
Trypsin 1	S01.151	209	Serine	14083	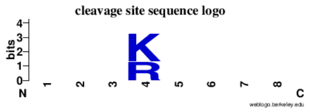
Thrombin	S01.217	204	Serine	185	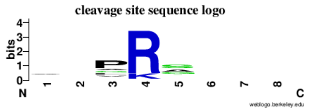
Plasmin	S01.233	1801	Serine	125	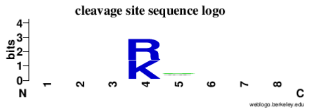
KLK 4	S01.251	4446	Serine	123	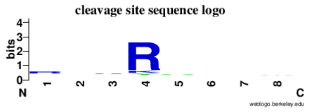
Glutamyl Peptidase I	S01.269	5115	Serine	1408	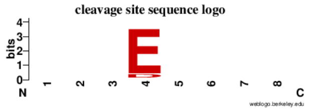
Lysyl Peptidase	S01.280	---	Serine	809	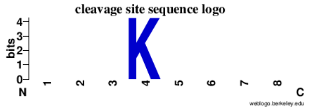
Lactocepin 1	S08.019	---	Serine	102	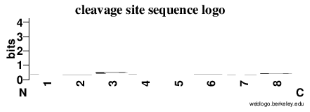
Kexin	S08.070	---	Serine	197	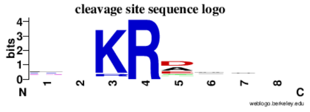
Furin	S08.071	2611	Serine	179	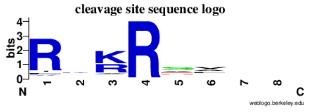
PCSK2 Peptidase	S08.073	2433	Serine	202	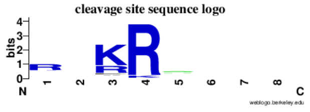
PCSK4 Peptidase	S08.074	4861	Serine	103	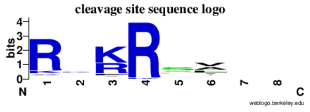
PCSK6 Peptidase	S08.075	2951	Serine	105	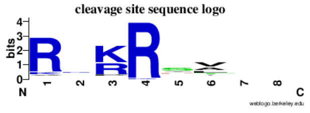

Protease	MEROPS	ChEMBL	Catalytic Type	Substrates	Sequence Logo
PCSK5 Peptidase	S08.076	2826	Serine	129	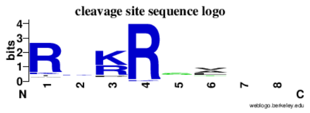
PCSK7 Peptidase	S08.077	2232	Serine	116	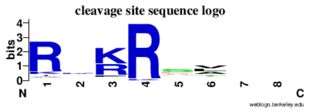
KPC2-type Peptidase	S08.109	---	Serine	115	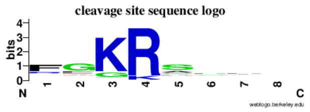
Lactocepin 3	S08.116	---	Serine	158	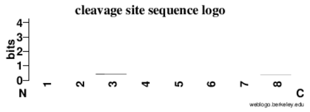
Signal Peptidase 1	S26.001	4470	Serine	342	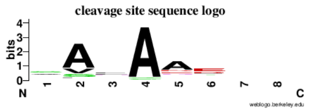
Signalase 21kDa	S26.010	---	Serine	363	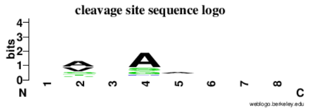
Signal Peptidase Complex	XS26.001	---	Complex	1880	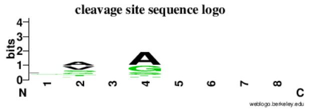

Pockets 1-8 correspond to P4...P1,P1'...P4' respectively
